# Antifungal activity of the repurposed drug disulfiram against *Cryptococcus neoformans*

**DOI:** 10.3389/fphar.2023.1268649

**Published:** 2024-01-11

**Authors:** Min Peng, Chen Zhang, Yuan-Yuan Duan, Hai-Bo Liu, Xin-Yuan Peng, Qian Wei, Qi-Ying Chen, Hong Sang, Qing-Tao Kong

**Affiliations:** ^1^ Department of Dermatology, Affiliated Jinling Hospital, Medical School of Nanjing University, Nanjing, China; ^2^ Affiliated Hospital for Skin Diseases, Chinese Academy of Medical Sciences, Nanjing, China

**Keywords:** *Cryptococcus neoformans*, disulfiram, drug repurposing, antifungal, drug resistance

## Abstract

Fungal infections have become clinically challenging owing to the emergence of drug resistance in invasive fungi and the rapid increase in the number of novel pathogens. The development of drug resistance further restricts the use of antifungal agents. Therefore, there is an urgent need to identify alternative treatments for *Cryptococcus neoformans* (*C. neoformans*). Disulfiram (DSF) has a good human safety profile and promising applications as an antiviral, antifungal, antiparasitic, and anticancer agent. However, the effect of DSF on Cryptococcus is yet to be thoroughly investigated. This study investigated the antifungal effects and the mechanism of action of DSF against *C. neoformans* to provide a new theoretical foundation for the treatment of *Cryptococcal* infections. *In vitro* studies demonstrated that DSF inhibited *Cryptococcus* growth at minimum inhibitory concentrations (MICs) ranging from 1.0 to 8.0 μg/mL. Combined antifungal effects have been observed for DSF with 5-fluorocytosine, amphotericin B, terbinafine, or ketoconazole. DSF exerts significant protective effects and synergistic effects combined with 5-FU for *Galleria mellonella* infected with *C. neoformans*. Mechanistic investigations showed that DSF dose-dependently inhibited melanin, urease, acetaldehyde dehydrogenase, capsule and biofilm viability of *C. neoformans*. Further studies indicated that DSF affected *C. neoformans* by interfering with multiple biological pathways, including replication, metabolism, membrane transport, and biological enzyme activity. Potentially essential targets of these pathways include acetaldehyde dehydrogenase, catalase, ATP-binding cassette transporter (ABC transporter), and iron-sulfur cluster transporter. These findings provide novel insights into the application of DSF and contribute to the understanding of its mechanisms of action in *C. neoformans*.

## 1 Introduction

Fungal infections are clinically challenging owing to the evolution of drug-resistant strains, emergence of new pathogens, prolonged treatment times, and limited antifungal treatment options (Wiederhold, 2017). *Cryptococcus neoformans* (*C. neoformans*) is a widespread opportunistic pathogenic fungus found in the environment. *C. neoformans* can enter the body through the respiratory tract, causing pulmonary infections and fatal meningitis, resulting in approximately 223,100 *Cryptococcal* meningitis cases and 181,100 fatalities annually (Rajasingham et al., 2017). As the coronavirus (CoV) disease 2019 pandemic has spread worldwide, cases of co-infection with *C. neoformans* and severe acute respiratory syndrome (SARS)-CoV-2 have been reported. These patients had high 30-day mortality rate (Bhatt et al., 2021; Chong et al., 2021; Mina et al., 2022). *Cryptococcus spp*. are susceptible to three classes of antifungal agents: polyenes, fluorocytosines, and azoles (Bermas and Geddes-McAlister, 2020). Nevertheless, the polyene amphotericin B (AmB) is susceptible to nephrotoxicity, anemia, and electrolyte imbalances (Klepser, 2011). Hepatotoxicity, gastrointestinal issues, and bone marrow suppression are common adverse effects of 5-fluorocytosine (5-FC), to which *C. neoformans* is frequently resistant (Klepser, 2011). Fluconazole (FLC) is an antifungal agent that plays a critical role in the initial treatment of *Cryptococcal* meningitis. However, its widespread use has resulted in frequent and persistent drug resistance (Kneale et al., 2016). Alternative treatment options for *C. neoformans* are urgently required considering the development of drug resistance, which severely restricts the use of antifungal medications.

Because fungi share organelles and metabolic pathways with human hosts, several antifungal medications can also harm humans. The development of antifungal drugs has presented significant challenges in the search for drugs with selective toxicity to fungi, but non-toxic or causing little harm to humans (Su et al., 2018). Toxicological and pharmacokinetic data have been established for conventional medications (Chong and Sullivan, 2007). Repurposing these medications for fungal therapy, or combining them with available antifungal drugs, is a promising strategy. For instance, the anticancer drug bortezomib inhibits the ubiquitin–proteasome pathway and capsule production in fungi (Geddes et al., 2016). Sertraline, an antidepressant, exerts antifungal effects when administered alone or in combination with FLC (Treviño-Rangel et al., 2016).

Disulfiram (DSF), often known as antabuse, is a safe dithiocarbamate well-tolerated by humans (Wright and Moore, 1990). Since 1951, when DSF was certified by the Food and Drug Administration (FDA) as an alcohol withdrawal drug, it has been used clinically for over 70 years. DSF has effective antiviral (Lee et al., 2016), antifungal (Mukherjee et al., 2006), antiparasitic (Peniche et al., 2015), and anticancer (Lu et al., 2022) properties. There are several mechanisms by which DSF inhibited bacterial growth and DSF can overcome multiple resistance barriers (Phillips et al., 1991; Thakare et al., 2019). DSF inhibits activities of many proteins, including aldehyde dehydrogenase (ALDH) (Velasco-García et al., 2003), metallo-β-lactamase (Chen et al., 2020), P-glycoprotein (P-gp), deoxyribonucleic acid (DNA)-methyltransferases, DNA polymerase (Sharma et al., 2016; Tesson et al., 2017), and phosphoglycerate dehydrogenase (Locasale et al., 2011). Disulfiram derivatives have been shown to exhibit the inhibitory effect against inflammatory scalp diseases by inhibiting carbonic anhydrase activity of *Malassezia*, which was an important causative pathogen for inflammatory dandruff (Vullo et al., 2017). DSF also inhibits the growth of *Pythium insidiosum* (Krajaejun et al., 2019). Additionally, it demonstrates significant antifungal activity against specific yeast strains, *Aspergillus*, and *Candida albicans* (Harrison et al., 2007; Khan et al., 2007). By inhibiting the function of multidrug transporter like Cdr1p, which has been shown to play a key role in azole resistance, DSF can increase the drug sensitivity of *Saccharomyces cerevisiae* (Shukla et al., 2004). Furthermore, due to its ability to curtail superoxide dismutase activity in *C*. *albicans* biofilm, the combination of DSF derivatives and AmB collaborates to limit biofilm activity (De Brucker et al., 2013). However, studies examining the effects of DSF on *C. neoformans* are still scarce.

In this study, we designed DSF sensitivity assays for 48 *Cryptococcus* spp. from different sources and performed combination susceptibility tests using common antifungal drugs. *Galleria mellonella* (*G. mellonella*) infected with *C. neoformans* was used to evaluate the *in vivo* antifungal activity of DSF. Mechanistically, we examined how DSF affects biofilm viability and the production of essential virulence components (melanin, urease, and capsule) in *C. neoformans*. Additionally, in combination with reference transcriptome sequencing, the molecular docking of DSF to protein targets was performed to investigate the mechanisms of the pathways and potential targets.

## 2 Materials and methods

### 2.1 Strains, culture, and reagents

Forty-two strains of *Cryptococcus* spp. were obtained from the strain banks of the Jinling Hospital (Nanjing, China). *Candida parapsilosis* (American Type Culture Collection 22019) was used as the quality control strain. The 5-FC stock solutions (6.4 mg/mL) were prepared from sterile ultrapure water. The FLC (6.4 mg/mL), ketoconazole (KET) (1.6 mg/mL), terbinafine (TRB) (1.6 mg/mL), AmB (1.6 mg/mL), and DSF (12.8 mg/mL) stock solutions were prepared from dimethyl sulfoxide (DMSO) and stored at −80°C. All reagents were purchased from Aladdin Biochemical Technology (Shanghai, China).

### 2.2 *In vitro* determination of minimum inhibitory concentrations of planktonic cells

Fungal solutions and drug susceptibility plates were prepared using the microdilution method in Roswell Park Memorial Institute (RPMI) 1,640 medium according to document M27-A4 published by the Clinical and Laboratory Standards Institute (CLSI). The final concentration of the fungal solution was adjusted to 5 × 10^3^ colony forming unit (CFU)/mL. One hundred microliters of RPMI 1640 and the drug were added to all wells of the plate. Through serial dilution at a ratio of two, concentrations from 256 to 0.5 μg/mL were obtained (DSF as example) and made in each plate, from column 1 to 10. The 11th column was reserved for the microorganism growth positive control. Then, 100 μL of the fungal suspension was added to each well. The assay was performed in triplicate and incubated at 35°C for 72 h. The MIC was defined as the lowest concentration that could inhibit fungal growth (Al-Odaini et al., 2021). The prepared drug-sensitive 96-well plates (U-shaped) were incubated at 35°C for 72 h. Each drug’s minimum inhibitory concentrations (MIC) and minimal fungicidal concentration (MFC) values against *Cryptococcus* were determined using a microplate reader (Thermo Fisher Scientific, United States) by combining the visual/microscopic reading and the optical density at 600 nm.

### 2.3 Combination susceptibility test using the checkerboard method

The gradient multiplicative dilution method was used to create a combined drug sensitivity plate (de Oliveira et al., 2020), and strain B3501 was used in this study. Considering the differences in chemical properties and cellular targets between DSF and other antifungal agents, both the Bliss synergistic score (https://synergyfinder.fimm.fi) and fractional inhibitory concentration index (FICI) based on Loewe’s summation theory were used to explain the drug-drug interactions (de Oliveira et al., 2020).

### 2.4 *In vivo* survival assays

Using *C. neoformans* (H99)-infected *G. mellonella* as a model, 80 healthy larvae of uniform size (250–350 mg) were randomly selected. According to the clinically safe dose, DSF was prepared using phosphate-buffered saline (PBS) (containing 10% DMSO), and the therapeutic concentration (500 mg/70 kg) was determined. The larvae were divided into four groups (10 larvae per group): infected group, the (post-infection) DSF treatment group, DSF control group, and PBS (10% DMSO) control group. The infected and (post-infection) DSF treatment group were injected with 10 μL of H99 fungal solution. The DSF and PBS groups were injected with 10 μL of PBS (pH 7.4). Two hours later, the infected group and the PBS control group were injected with 10 μL of PBS (containing 10% DMSO) and the (post-infection) DSF treatment group and the DSF control group were injected with 10 μL of DSF treatment solution. Fatality was defined as the failure to respond to a light touch. Inoculation was achieved by injecting the inoculum into the hemocoel of the second left foot (Sudan et al., 2013). The fresh H99 fungal solution was incubated overnight in a shaker at 30°C (180 rpm) and centrifuged for 5 min at 2,800 × g (Thermo Fisher Scientific). The fungal precipitate was washed thrice with sterile PBS (pH 7.4). After resuspension, the fungal solution was counted using a cell-counting plate and diluted to 2 × 10^6^ and 2 × 10^7^ CFU/mL with PBS. *G. mellonella* were incubated in the dark at 37°C for 5–7 days, and their survival was monitored every 24 h.

The *in vivo* antifungal activities of 5-FU, DSF and their combination were evaluated in a higher concentration model (each injected with 10^6^ CFU H99 in 10 μL PBS). Through repeated preliminary experimentation, the concentrations of 5-FU at 1.25 mg/kg combined with DSF at 3.5 mg/kg were used for treated. Groups treated only with 5-FU or DSF alone were dosed at 2.5 mg/kg and 7.1 mg/kg, respectively. The volume of solvent and the method of preparation were the same for all groups during the treatment.

### 2.5 Determination of fungal burden

An additional 160 healthy larvae of uniform size (250–350 mg) were randomly selected. Four groups of randomly selected larvae (20 for each group) were established as described above and incubated at 35°C for 4 days (Kong et al., 2020). During incubation, five larvae from each group were randomly selected daily on days 1–4. A high-speed homogenizer (Powteq GT300, Grinder Instrument, Beijing, China) was used to thoroughly grind the larvae for 30 min (1,600 rpm) after adding 0.7 mL PBS and sterile magnetic beads (3 mm, Beyotime, Haimen, China). We used PBS to dilute the homogenate in a gradient and inoculated 5 μL of samples with different concentrations on Sabouraud dextrose agar (SDA) at 37°C for 48 h.

### 2.6 Histological examination

Histological analysis was performed to observe the effects of DSF on *G. mellonella* larvae infected with *C. neoformans* (H99). Another 40 healthy larvae of uniform size (250–350 mg) were randomly selected. Four groups of randomly selected larvae were injected with fungal inocula and DSF, as described above. Three *G. mellonella* in each group were taken for pathological section (20 μm) and periodic acid-Schiff staining (Solarbio, Beijing, China). The staining results were observed under a light microscope (Zeiss Axioscope 5, ZEISS, Germany) at different magnifications (Sangalli-Leite et al., 2016).

### 2.7 Melanin production assay

Fresh H99 (*C. neoformans* var. *grubii*), ZYB24 (*C*. *gattii*), B3501 (*C. neoformans* var. *neoforman*), and D2A (*C. neoformans* var. *neoforman*) solutions were washed thrice with sterile PBS to reduce the melanin background. A simplified dopa-asparagine agar (with MgSO_4_ at 10 mM, KH_2_PO_4_ at 29.4 mM, glycine at 13 mM, vitamin B1 at 3.0 µM, L-asparagine at 7.6 mM, and L-dopa at 1 mM) was prepared (Teymuri et al., 2021). Different concentrations of DSF (range 0.5–8 μg/mL) were applied to dopa-asparagine agar before being inoculated with 10 μL of the fungus solution protected from light for 2–7 days at 35°C. The inoculum concentration was 1 × 10^7^ CFU/mL. Images of the plates were captured daily (García-Rodas et al., 2015).

### 2.8 Urease activity assay

Urea slant agar mediums containing different concentrations of DSF (0.5–8 μg/mL) were prepared. Agar was prepared with a urease agar base (Hi-tech, Qingdao, China) and sterile urea solution according to Christensen’s formula (Christensen, 1946). The mediums were inoculated with fresh fungal solutions (10 μL), and color changes were observed for 2–10 days. Urease activity judgment index: The medium turned completely purple-red (+++) or pink (++); only the colony and surroundings turned pink (+); and no color change (−). Rapid urea broth (RUH broth), developed by Roberts (Roberts et al., 1978) and adapted by Kwon-Chung (Kwon-Chung et al., 1987) was used to detect urease activity for quantitative statistics. Fungal suspension (100 μL) was mixed with an equivalent amount of RUH broth (two times concentration) and shaken for 10 h at 37°C. The absorbance (560 nm) of the supernatant of equal fungal amounts was measured using a microplate reader (Feder et al., 2015).

### 2.9 Determination of capsule size

The H99 and B3501 strains were inoculated into SDA and capsule induction medium [10% SDA, 3-(N-morpholino) propanesulfonic acid (MOPS)] at a 5 × 10^5^ CFU/mL concentration (Zaragoza and Casadevall, 2004). Positive control and DSF groups (MICs concentration) were shaken for 18–24 h at 37°C. The mixture of fungal solution (4 μL) and India ink (6 μL, Yuanye Biotechnology, Shanghai, China) was added dropwise on a slide and photographed by the MShot Image Analysis System.

### 2.10 Biofilm assay

Fresh H99 and B3501 fungal solutions were resuspended in RPMI 1640 (MOPS, pH 7.4) at a concentration of 1 × 10^7^ cells/mL. To investigate the degradation effect of DSF on biofilms, two hundred microliters of fungal broths of H99 or B3501 were added to each well of the sterile 96-well plates (Clear polystyrene microplates, Corning, NY, United States) and incubated at 30°C for 2 h. The solutions were aspirated and softly washed with PBS before being replaced with RPMI 1640 (Sigma, Cleveland, United States) of different DSF gradient concentrations (0.25–128 μg/mL) and then incubated for 48 h. Furthermore, the medium and non-adherent cells were discarded, and the biofilms were stained with 0.1% crystal violet (Yuanye Biotechnology, Shanghai, China). Following the washing and drying of the biofilm, 150 μL of 50% acetic acid was added to each well. The absorbance was measured at 530 nm using a microplate reader (Thermo Fisher Scientific) to determine the level of biofilm degradation (Segev-Zarko and Shai, 2017; Song et al., 2019; Huang et al., 2023). The data for the drug-treated group was normalized relative to the control group.

Cellular activity within the biofilm was quantified based on the capacity of living cells to reduce the methyl thiazolyl tetrazolium (MTT, Beyotime, Haimen, China) yellow dye to purple formazan. The new batch of biofilm was incubated (1 × 10^6^ cells/mL) for 48 h before being replaced with RPMI 1640 containing DSF for another 24 h. Afterwards, the MTT formazan product was dissolved in 150 μL DMSO and the absorbance was measured using the multiplate reader (da Silva et al., 2016; Huang et al., 2023).

### 2.11 Transcriptome sequencing and real-time quantitative PCR (RT-qPCR)

The PBS-washed H99 solution was inoculated in fresh yeast extract peptone dextrose medium at a final concentration of 1 × 10^6^ CFU/mL for 4 h. Control and DSF (4 μg/mL) groups were prepared. Total ribonucleic acid (RNA) was extracted using the TRIzol reagent (Invitrogen, Carlsbad, CA, United States) and evaluated using a NanoDrop 2000 spectrophotometer (Thermo Scientific). RNA integrity was assessed using the Agilent 2,100 Bioanalyzer (Agilent Technologies, Santa Clara, CA, United States). Libraries were then constructed using the VAHTS Universal V6 RNA sequencing (RNA-seq) Library Prep Kit and sequenced on an Illumina NovaSeq 6000 platform. Raw reads in fastq format were first processed using fastp (Chen et al., 2018), and clean reads were mapped to the reference genome using HISAT2 (Kim et al., 2015). The differential expression (DE) analysis was performed using DESeq2 (Love et al., 2014). DE genes (DEGs) were identified between DSF-treated and control groups. Based on the hypergeometric distribution, Gene Ontology (GO) and Kyoto Encyclopedia of Genes and Genomes (KEGG) pathway enrichment analyses of the DEGs were performed to screen for significantly enriched terms using R (v 3.2.0).

The expression of mRNAs was verified by RT-qPCR (Yi et al., 2022). RNA was used to synthesize cDNA using the PrimeScript RT Reagent Kit (Vazyme, Nanjing, China). RT-qPCR was performed with the help of SYBR GREEN PCR MasterMix (Vazyme) and an Applied Biosystems 7500 real-time machine. Actin was used as a housing-keeping gene. The primer sequences are detailed in Supplementary Table SA5.

### 2.12 Homology modeling of *C. neoformans* proteins and molecular docking of DSF

The amino acid sequences of DEGs were imported into the Protein Data Bank (PDB) database. Based on Sequence Identity and E-Value, as well as functional and species annotations, homologous proteins with great matches were chosen, and their crystal structures were downloaded. Structural information of the DSF molecules was obtained from the PubChem database. The corresponding protein structures of H99 predicted using AlphaFold were obtained from the UniProt database. The structures of the *Cryptococcus* proteins and homologous proteins were imported into the PyMol software, and the alignment command was executed. After executing the Autogrid program in the AutoDockTools software for acceptors and ligands, the AutoDock Vina program was run to perform calculations to obtain multiple docking models. Finally, the structure of the ligand-acceptor complex was imported into Discovery Studio 2021 to visualize the chemical bonds.

### 2.13 ALDH activity assay

To analyze acetate production by *C. neoformans*, an ALDH activity assay was performed (Ammar, 2019). Fresh H99 strains cultured overnight in yeast extract peptone dextrose medium were expanded to 5 mL of yeast nitrogen base (Thermo Fisher Scientific) medium with 2% glucose at a concentration of 1 × 10^4^ CFU/mL. The control and DSF (2 μg/mL) groups were incubated at 30°C for 48 h. The final concentration of each fungal solution was adjusted to the same level (OD_600_ = 0.65) at the time of measurement and was centrifuged at 10,000 × g (Thermo Fisher Scientific). Once all supernatants were collected, the concentration of acetic acid, a metabolite catalyzed by ALDH (Pronk et al., 1996), was measured using an ACETIC ACID Kit (K-ACET, Megazyme, Ireland).

### 2.14 Statistical analysis

All experiments were performed in triplicate unless specified otherwise. Statistical analysis was performed using SPSS Statistics 23.0. The statistical procedure utilized was the Student’s t-test. Survival curves were plotted using the Kaplan–Meier method and statistical analysis was performed using the log-rank test. Each experiment was performed in triplicate and repeated at least thrice. The value *p* < 0.05 was considered significantly different (*p* < 0.05 for *, *p* < 0.01 for **, and *p* < 0.001 for ***).

## 3 Results

### 3.1 DSF demonstrated broad-spectrum and strong susceptibility to *C. neoformans* and *C. gattii in vitro*

**FIGURE 1:**
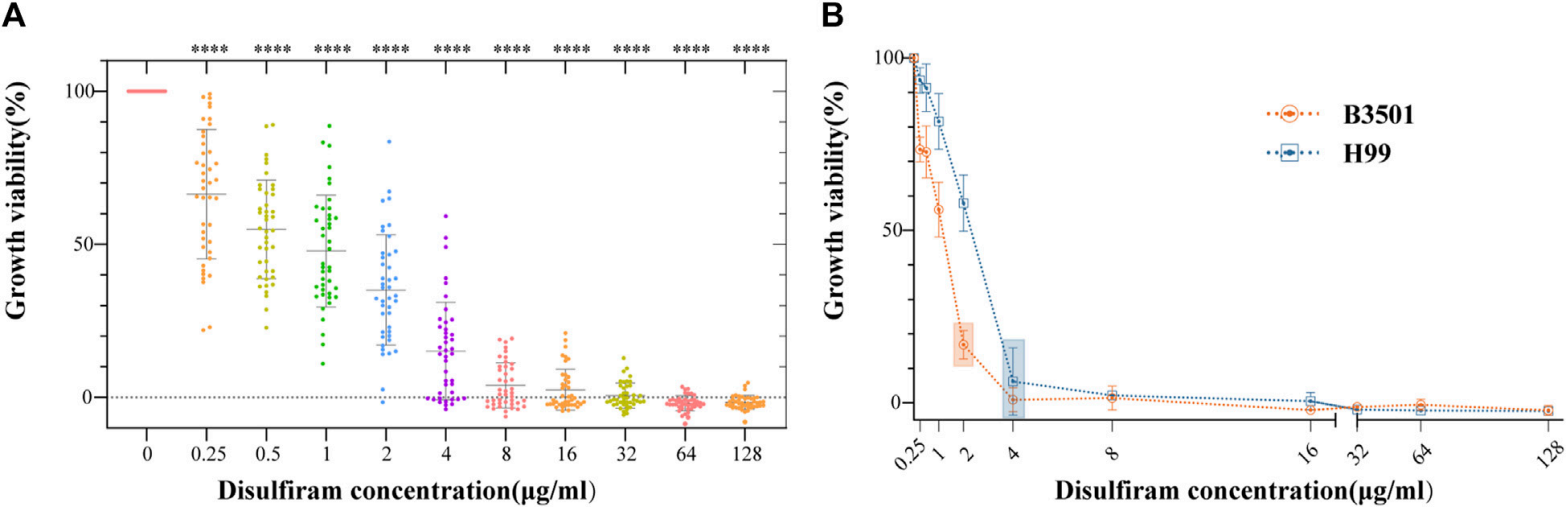
*In vitro* sensitivity assays of DSF against 42 strains of *Cryptococcus*. **(A)** Growth viability (%) of different *Cryptococcus* serotypes exposed to different DSF concentrations (ranging from 0 to 128 μg/mL). ****p < 0.0001, vs. positive controls. **(B)** Growth of B3501 and H99 sensitive to DSF at 2 and 4 μg/mL (boxed area), respectively.

The 42 *Cryptococcus* isolates in this study, covering the four serotypes A, B, C, and D, showed MICs ranging from 1 to 8 μg/mL (Figure 1A). As shown in Supplementary Table SA1, the FLC MICs for these strains ranged from 0.5 to 16.0 μg/mL. These findings suggest that DSF efficiently suppressed non-FLC-resistant *Cryptococcus* strains of various serotypes. The results for the quality control strain ATCC 22019 were in accordance with those of CLSI-M27-A4. The MIC of DSF was 2 μg/mL against B3501 and 4 μg/mL against H99 (Figure 1B).

### 3.2 DSF potentiated the activity of clinical antifungal drugs

**FIGURE 2:**
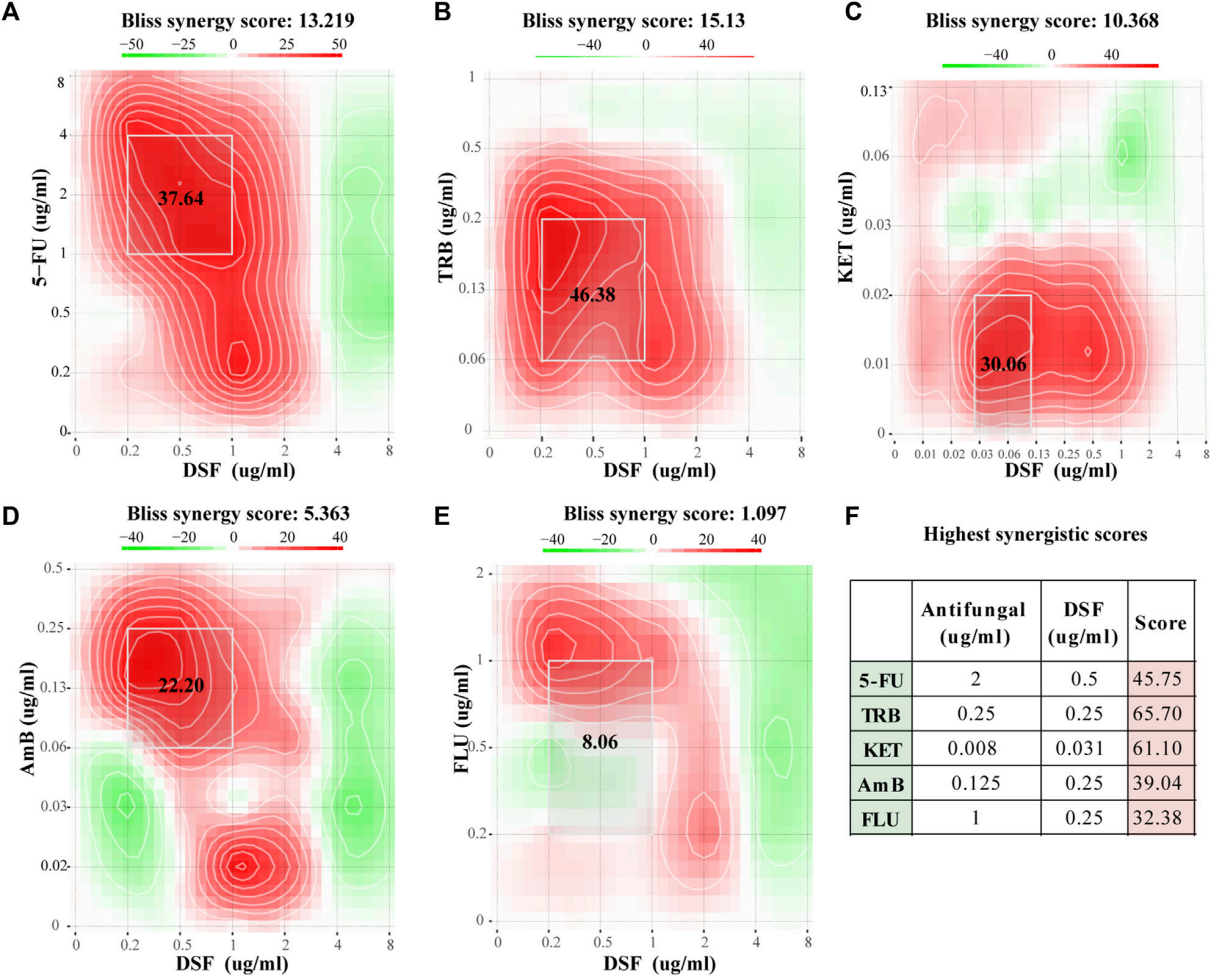
Checkerboard assay results for DSF against *C. neoformans*. **(A–E)** Bliss scores combined with 5-FC, TRB, KET, AmB, and FLC. **(F)** Highest score for each group. Scores less than −10 indicate that the interaction between the two drugs was antagonistic. Scores from −10 to 10 suggest an additive effect, and scores above 10 indicate synergism.

Figures 2A–E show the Bliss scores for the efficacy of DSF in combination with the common antifungal agents 5-FC, TRB, KET, AmB, and FLC against *C. neoformans*. For instance, when 5-FU and DSF were combined at 2 and 0.5 μg/mL, respectively, a high synergistic score of 45.75 was obtained (Figure 2F). Synergistic activity with DSF was observed in a broader concentration range of 5-FC, TRB, KET, and AmB. For instance, in the combined concentration range of DSF from 0.25 to 1 μg/mL and 5-FC from 1 to 4 μg/mL, a Bliss score of 37.64 was calculated. The TRB, KET, AmB, and FLC scores were 46.38 (synergistic), 30.06 (synergistic), 22.20 (synergistic), and 8.06 (no interaction), respectively. This was in accordance with the findings obtained from the FICI scores (Supplementary Table SA2).

### 3.3 DSF protected *G. mellonella* from H99 infection *in vivo*

**FIGURE 3:**
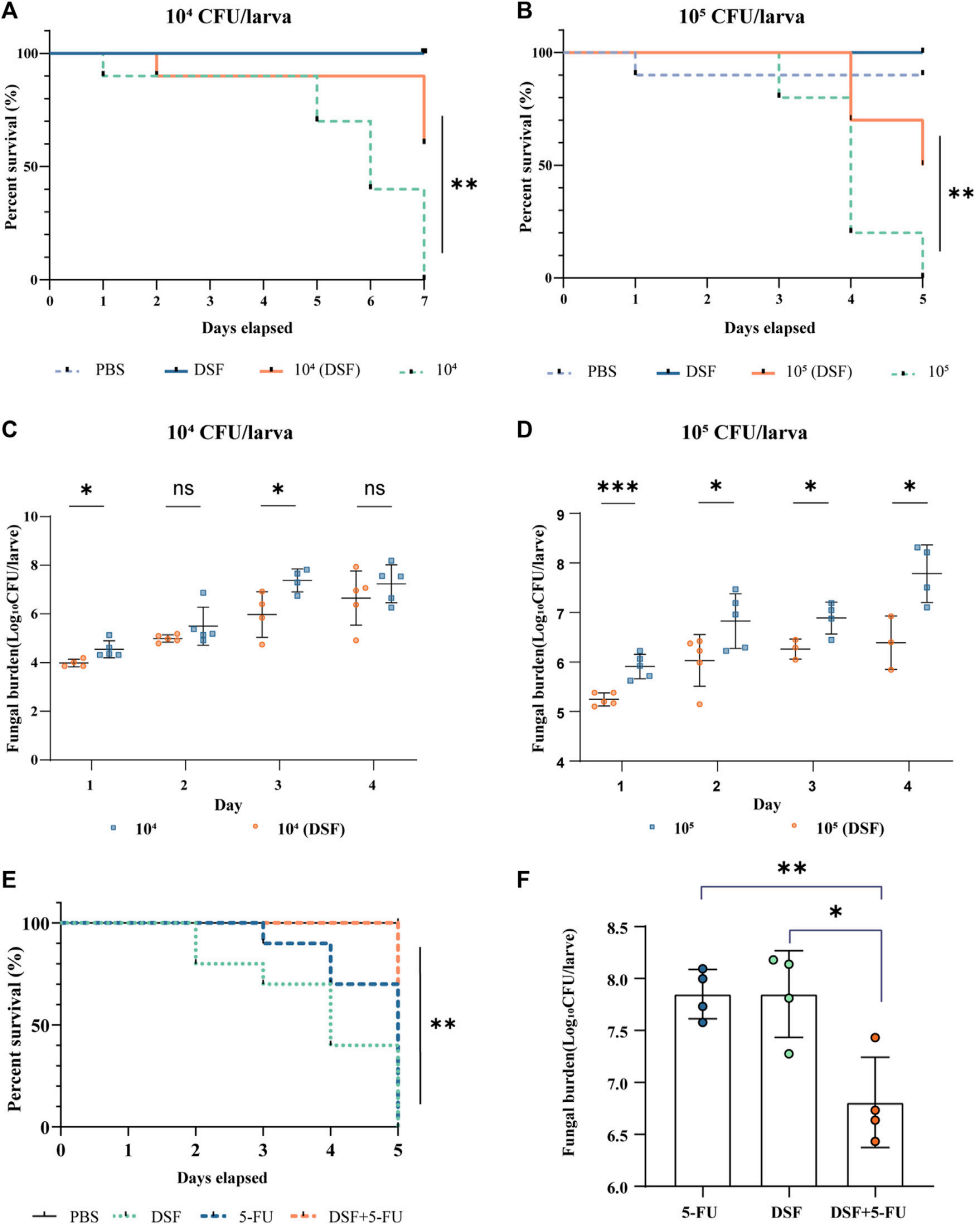
Protective effects of DSF against H99-infected *G. mellonella*. **(A,B)** Survival curves at different infection concentrations. **(C,D)** Fungal burden of infected larvae. The treatment groups were administered DSF (7.1 mg/kg) at 3 h after infection. The PBS and DSF groups were used as negative controls. Compared with the infected group, *p < 0.05, **p < 0.01, and ***p < 0.001. **(E)** Survival curves of different treatments on infected larvae. **(F)** Fungal burden of different treatments on infected larvae. Each group was treated with either PBS (10% DMSO), 5-FU (2.5 mg/kg), DSF (7.1 mg/kg), or 5-FU (1.25 mg/kg) plus DSF (3.5 mg/kg). *p < 0.05, **p < 0.01, compared with the 5-FU-treated group. 5-FU, 5-fluorocytosine; DSF, disulfiram.

In the present study, *G. mellonella* larvae were infected with strain H99 as an experimental model and larval mortality was observed daily for 4–7 days. The larval survival rate on day 7 was 0% in the infected groups at a concentration of 1 × 10^4^ CFU/mL (10^4^-infected groups) and 60% in the DSF-treated group (Figure 3A), with a significant difference between the groups (*p* < 0.01). The survival rate of the 10^5^-infected group was 0% on day 5 and increased to 50% after DSF treatment (Figure 3B, *p* < 0.01). This indicates that DSF has antifungal efficacy *in vivo* and significantly improves the survival rate of H99-infected larvae.

### 3.4 Determination of fungal burden

Five larvae per group were selected, homogenized, inoculated, and counted daily using fungal load analysis. The fungal burden gradually increased with the duration of infection. The fungal burden was lower in the DSF-treated group than that in the respective infection groups at different H99 concentrations (Figures 3C, D). In particular, DSF treatment significantly reduced the fungal burden on the first day in the 10^5^-infected group (*p* < 0.001).

### 3.5 Histological examination

**FIGURE 4:**
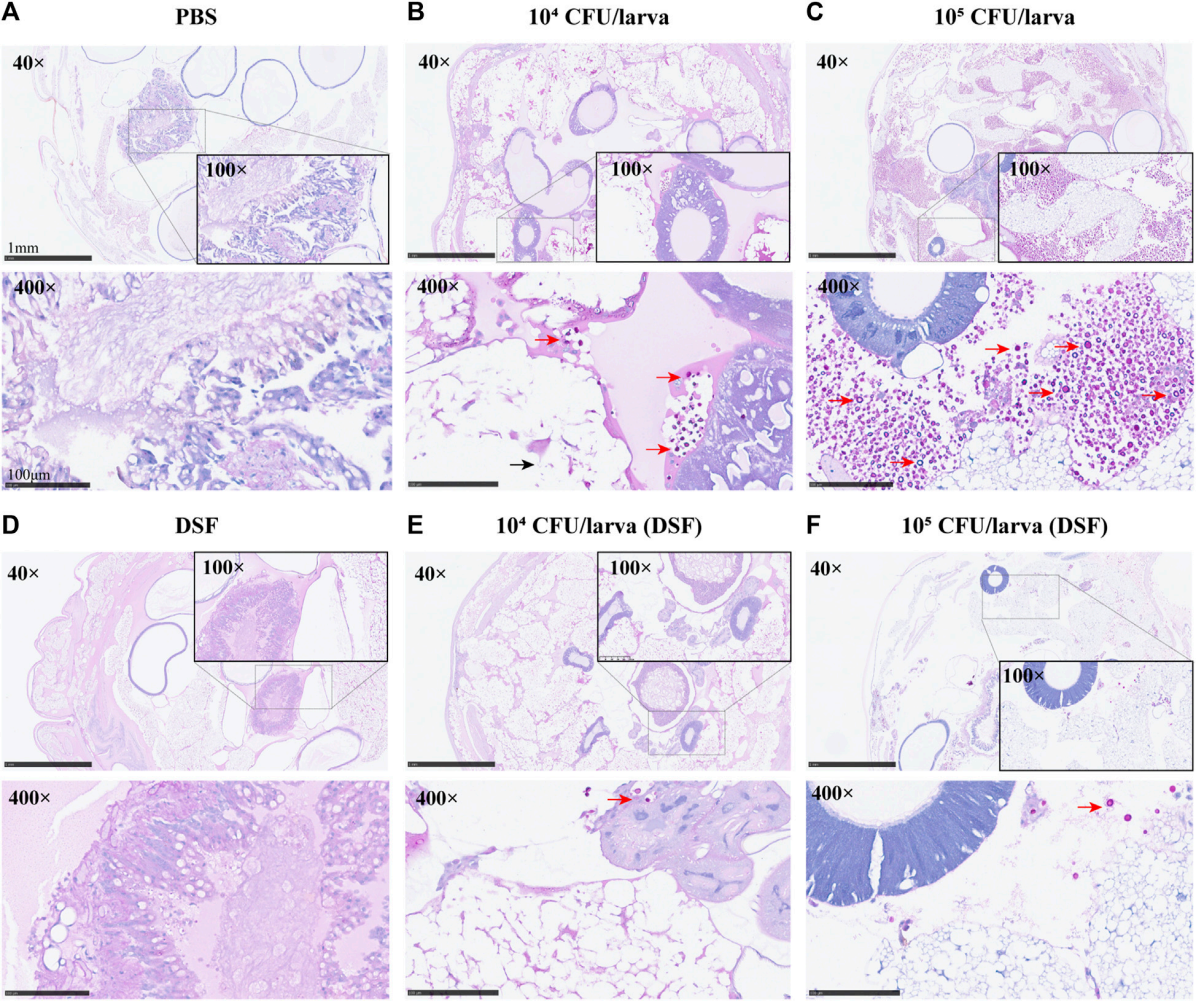
Histological examination (×40, ×100, and ×400) of *G. mellonella* larvae. **(A)** PBS (10% DMSO) control group. **(B,C)** Group infected with concentrations of H99 1 × 10^4^ or 10^5^ CFU/larva. **(D)** DSF control. **(E,F)** DSF-treated infected larvae group. Sections were stained with the PAS reagent; red arrows indicate *C. neoformans* and black arrows indicate tissue destruction.

On day 3, the protective effect of DSF on *G. mellonella* was observed by histopathology. The periodic acid-Schiff (PAS)-stained sections were photographed at different magnifications (×40, ×100, and ×400). Compared with the PBS and DSF control groups (Figures 4A, D), the infected larvae (Figures 4B, C) displayed extensive tissue destruction, with many dense capsule antigens stained in a typical purple-red color and surrounded by inflammatory cells. A significant reduction in the number of *C. neoformans* cells was observed in the DSF-treated groups (Figures 4E, F), and larval tissue damage was considerably reduced.

### 3.6 Synergistic effect of DSF combined with 5-FU against *C. neoformans in vivo*

The *in vivo* antifungal activities of 5-FU, DSF and their combination were evaluated in an infected *G. mellonella* model. The concentrations of 5-FU at 1.25 mg/kg combined with DSF at 3.5 mg/kg were used, showing a strong antifungal activity against H99 *in vivo*, compared with only use of 5-FU (2.5 mg/kg) or DSF (7.1 mg/kg). These concentrations of the drugs used in this experiment meet the requirements for safe human dosages. The survival assay (Figure 3E) showed that when DSF was added in combination with 5-FU, the survival rate of infected larvae of a higher concentration model was improved and the fungal burden in day 2 (Figure 3F) was significantly induced (*p* < 0.05). These results indicated that DSF in combination with 5-FU exhibited a significant synergistic antifungal effect against *C. neoformans in vivo*.

### 3.7 DSF inhibited melanin production by *Cryptococcus* spp. in a dose-dependent manner

**FIGURE 5:**
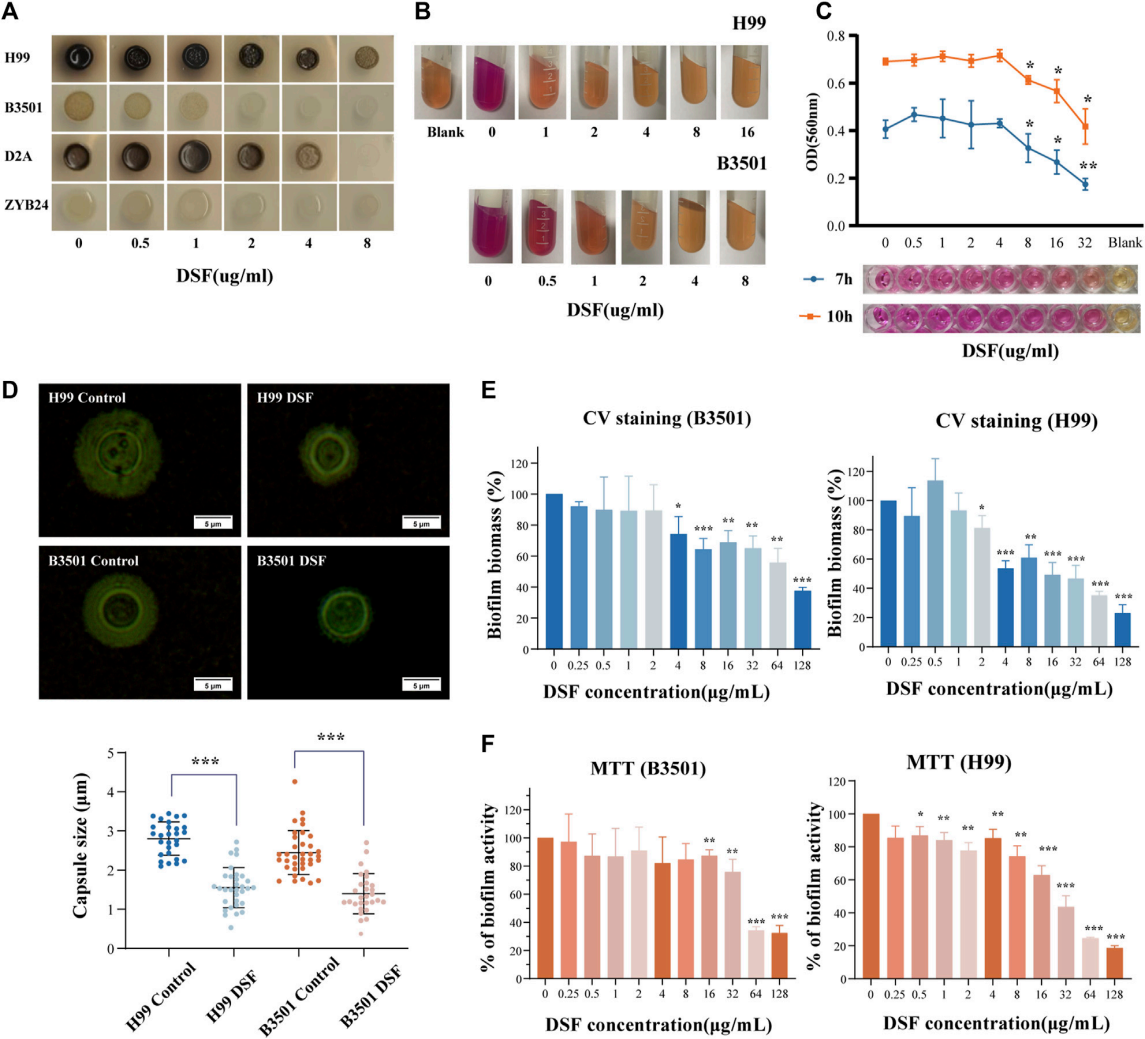
Effect of DSF on virulence factors of *C*. *neoformans*. **(A)** Melanin production on agar plates with different doses of DSF; **(B)** Urease activity on agar; **(C)** Variation in urease activity in RUH liquid medium. The rightmost well contained the RUH medium control group. **(D)** India ink staining after induction. Capsule thickness is defined as the distance between the cell wall and outer boundary of the capsule. **(E)** Biofilm degradation; **(F)** Viability of cells within the biofilm. *p < 0.05, **p < 0.01, ***p < 0.001, vs. controls.

*Cryptococcus* produces virulence factors, such as polysaccharide capsules, melanin, and extracellular enzymes (including phospholipases, proteases, and urease), and tolerates a 37°C environment (Alspaugh, 2015). These factors protect cells from desiccation and oxidative stress damage and disrupt the host immune response (Nosanchuk et al., 2015; Lee et al., 2019). We examined the melanin-producing ability of different serotypes of *Cryptococcus* on dopa-asparagine agar at 30°C (Figure 5A). Colonies grew at all time points, and the positive controls produced varying degrees of melanin. As the concentration of DSF gradually increased (0.5–8 μg/mL), the melanin production of each strain progressively decreased (from dark brown to coffee or from light coffee to white). This suggests that DSF exerts a dose-dependent inhibitory effect on the production of *Cryptococcal* melanin.

### 3.8 DSF inhibited the urease activity of *C. neoformans*

Once the urea substrate is broken down by urease, the alkaline product discolors the medium. The positive control exhibited a strong urease activity (+++). With increasing concentration of DSF in the medium (Figure 5B), no color change (−) occurred in all groups except for the 0.5 μg/mL and 1 μg/mL groups, which showed (++/+) changes. The modified RUH experiment monitored changes in urease activity in a shorter time (Figure 5C) and controlled the interference of the fungal load. When DSF was increased to 8 μg/mL, the activity was obviously reduced compared to that in the non-dosed group (*p* < 0.05). This showed that *C. neoformans* urease activity was inhibited by DSF as the drug concentration increased.

### 3.9 DSF restricted capsule formation of *C. neoformans*

The capsules stained with ink were photographed and counted (Figure 5D). The capsule thickness was 2.803 ± 0.127 μm [*mean ±* standard deviation (*SD*)] in the H99-control group and 1.551 ± 0.513 μm in the H99-DSF group (*p* < 0.001 compared to the control). The capsule thickness was 2.448 ± 0.563 μm (*mean ± SD*) in the B3501-control group and 1.399 ± 0.512 μm in the B3501-DSF group (*p* < 0.001). The inhibition of capsule formation of *C. neoformans* by DSF contributed to the reduction in fungal resistance and invasiveness.

### 3.10 Biofilm inhibition effects of DSF

Biofilm increases the virulence of Cryptococcus and facilitates drug resistance. Biofilms were prepared using a fungal solution. After incubation period to form biofilms, we measured the optical density of each well and found consistent values. Compared to the control, H99 showed a statistical difference at the DSF concentration up to 2 µg/mL and B3501 at 4 µg/mL compared to the control (Figure 5E). This suggests that DSF had a degradation effect on the formed biofilm. Another group of prepared 48 h-biofilms was incubated for 24 h with DSF. The number of living cells within the biofilm decreased as drug concentration increased. Compared to the control, a statistical difference was observed at 0.5 µg/mL DSF concentration for H99 and at 16 µg/mL for B3501 (Figure 5F). The data for the drug-treated group was normalized relative to the control group.

### 3.11 DSF controlled pathways related to cell division and biological enzyme activity of H99

**FIGURE 6:**
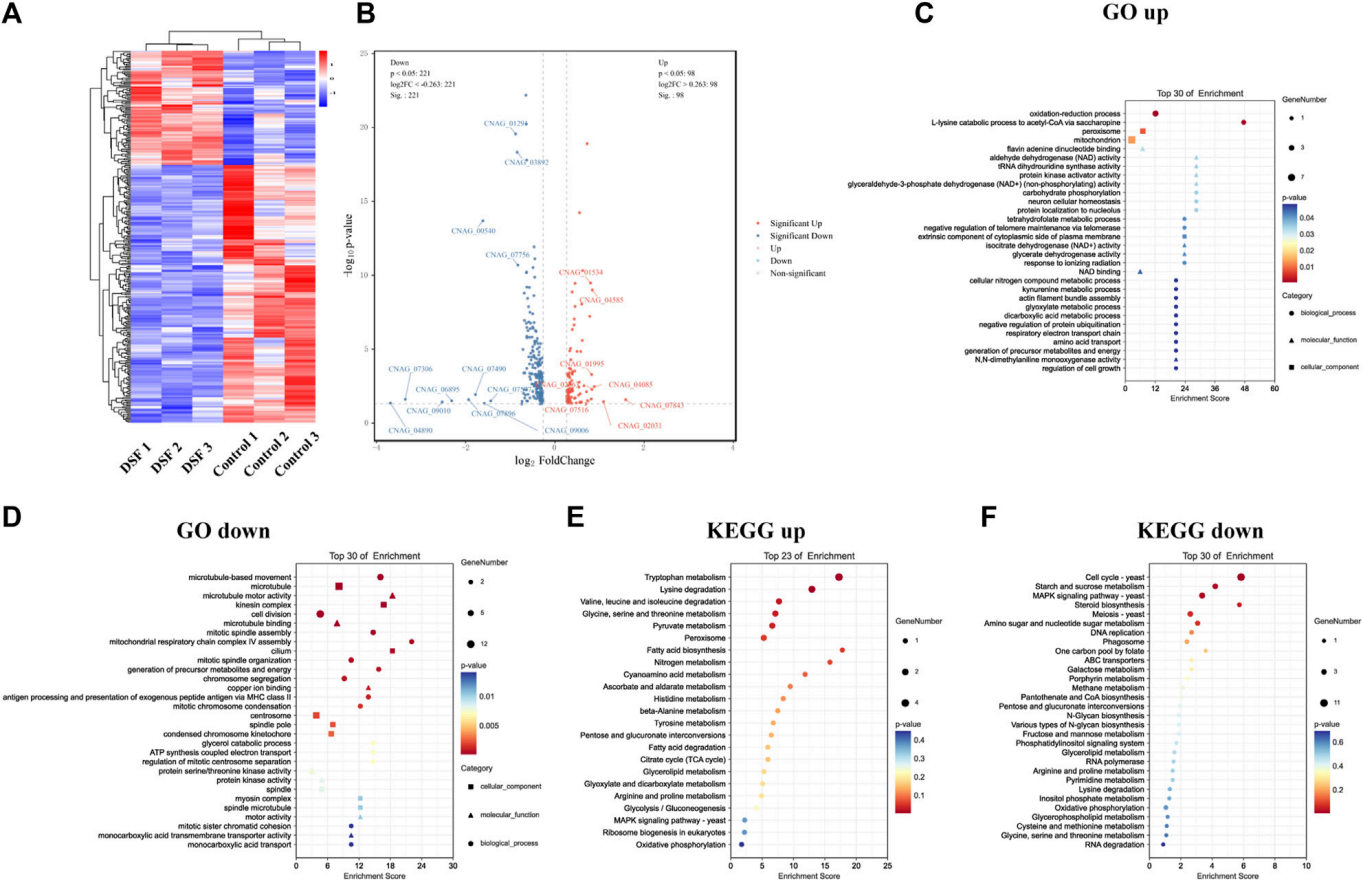
Transcriptome sequencing profile of H99 cells after DSF treatment. **(A)** Heat map of DEGs clustering. Red, highly expressed genes; blue, low-expression genes. **(B)** Volcanic map. Gray: non-significant DEGs; red and blue: significant DEGs. **(C,D)** Bubble map of GO enrichment. **(E,F)** Bubble map of KEGG enrichment.

To further investigate the mechanism of action of DSF against *C. neoformans*, we analyzed the regulation of the gene expression at the transcriptional level. A1-A3 (parallel replicates) were H99 control groups, and B1–B3 were H99 treated with 4 μg/mL of DSF. A total of 319 DEGs were identified. Cluster analysis showed that 98 DEGs were upregulated and 221 were downregulated in the DSF group compared to those in the control group (Figure 6A). The overall distribution of DEGs was observed in a volcano plot (Figure 6B).

Enrichment analysis was performed using the GO database, and 10 items with a respective number of upregulated (Figure 6C) and downregulated DEGs (Figure 6D) greater than or equal to two under subcategories were screened for bubble plots. DEGs were mainly enriched in the oxidation-reduction process (GO:0055114), peroxisomes (GO:0005777), mitochondria (GO:0005739), and nicotinamide adenine dinucleotide (NAD^+^) binding (GO:0051287). Downregulation was highly significant, with the DEGs mainly enriched in microtubule-based movements (GO:0099098), cell division (GO:0051301), microtubule motor activity (GO:0003774), protein serine/threonine kinase activity (GO:0004674), protein kinase activity (GO:0004672), and adenosine triphosphate hydrolase activity (GO:0042623). During this process, DSF regulates cell division, important biological enzyme activities, and reduction-oxidation (redox)-related pathways in *C. neoformans*.

### 3.12 DSF affected membrane transport function and multiple metabolic processes of H99

The KEGG database was also used for the enrichment analysis of DEGs, and 2,387 genes were annotated to 36 KEGG pathways (Figures 6E, F). The downregulated pathways were highly significant (Figure 6F) and involved cell growth and death, transport and catabolism, membrane transport, signal transduction, folding/sorting and degradation, amino acid metabolism, carbohydrate metabolism, energy metabolism, metabolism of cofactors and vitamins, and nucleotide metabolism. In conclusion, we found that DSF affects membrane transport function and multiple metabolic processes in *C. neoformans*. We verified the up and downregulation of eight DEGs (upregulated: *CNAG_04085*, *CNAG_01995*, *CNAG_04585*, *CNAG_02863*; downregulated: *CNAG_05752*, *CNAG_07756*, *CNAG_00540*, *CNAG_00735*) by q-PCR. The q-PCR data were in agreement with RNA-seq data (Supplementary Figure S2).

### 3.13 Homology modeling of ALDH (NAD^+^) and molecular docking of DSF

Based on the confirmed mechanism of DSF, we screened H99 functional proteins, including ALDH (NAD^+^), catalase (CAT), ABC multidrug transporter AFR2, iron-sulfur clusters transporter ATM1, based on the essential differential expression pathways suggested by RNA-seq. They serve as critical biological enzymes or transport-related proteins and are closely related to the growth, metabolism, virulence, drug resistance, and oxidation-reduction processes of *C. neoformans*. They were then docked to DSF ligands for calculations.

**FIGURE 7:**
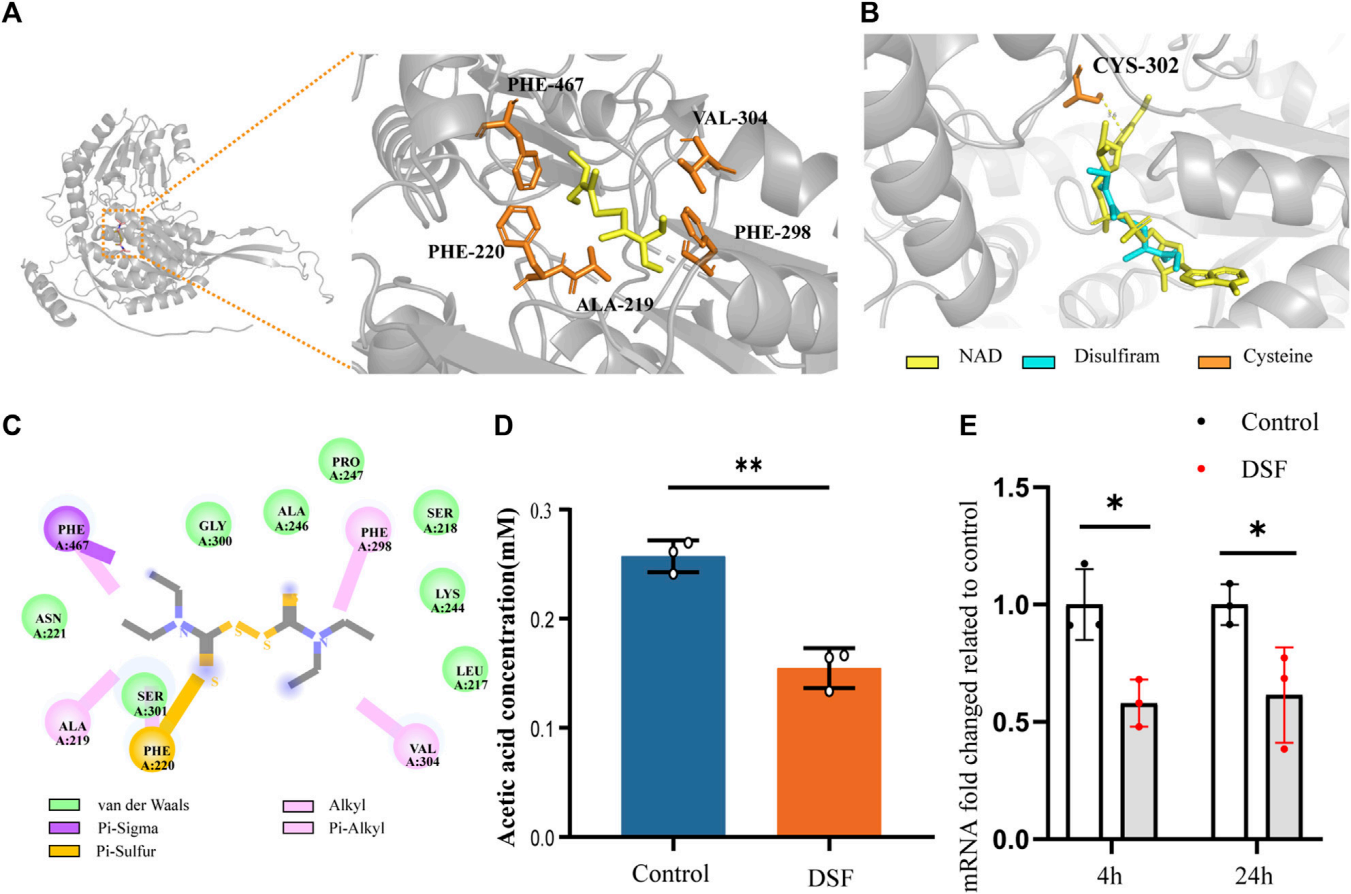
DSF disrupts acetic acid metabolism by affecting mRNA expression and protein structure of ALDH (NAD^+^) from *C*. *neoformans*. **(A)** The complexes ribbon structure and enlarged molecular docking images of the DSF ligand with predicted Alpha-Fold 3D structures of ALDH (NAD^+^) from H99. **(B)** The optimal DSF conformation containing coordinate information of the homologous protein structure. **(C)** 2D interactions between DSF and the predicted binding sites of H99 ALDH (i.e., conventional H-bond, carbon-hydrogen bond, alkyl or Pi-alkyl bond, Pi-sulfur bond, Pi-sigma bond, and Van der Waals bond). **(D)** Biochemical effects of DSF on ALDH in H99 cells. **(E)** RT-qPCR results of *ALDH* (*NAD* ^*+*^) of different time, *p < 0.05, **p < 0.01, vs. controls.

DSF was docked to the Alpha-Fold predicted structure of H99’s ALDH (NAD^+^) (UniProt ID: J9VME7, open reading form (ORF) name: CNAG_00735), with a complex binding affinity of −4.4 kcal/mol (Figure 7A). The optimal conformational affinity and root-mean-square deviation of the receptor pocket are shown in Supplementary Table SA3. In the ligand-receptor complex, ALDH interacted with DSF through five amino acid residues in the active pocket, including PHE-467, VAL-304, PHE-298, ALA-219, and PHE-220 (Figure 7C). The best-matched human protein for ALDH (NAD^+^) (PDB ID 4X0T) and matching parameters are shown in Supplementary Table SA4. By importing the optimal DSF docking conformation containing the coordinate information, we found that the position of the DSF molecule coincided with the active substrate NAD^+^ of ALDH (4X0T). Moreover, DSF was located at the center of four cysteine (Cys) residues, with the closest residue at position 302 (Figure 7B). Moreover, *ALDH* of H99 showed significant downregulation results (*p* < 0.05) in the KEGG Pathway significance analysis. The q-PCR results (Figure 7E) also verified this result.

### 3.14 DSF inhibited the ability of *C. neoformans* to metabolize aldehydes

ALDH, an essential metabolic enzyme in the yeast fungus, transforms harmful aldehyde metabolites into acetic acid, which is then excreted. *C. neoformans* isolated from the lungs of infected mice expresses high levels of ALDH transcripts, and ALDH expression is important for protecting C*ryptococcus* cells from phagocytosis or destruction by macrophages after engulfment (Hu et al., 2008). ALDH knockout mutants have partially compromised cell walls and exhibit reduced recovery after phagocytosis and significantly reduced virulence in *G. mellonella* infection model (Ammar, 2019). To determine whether DSF interferes with the catalytic pathway involving ALDH, we assayed the biochemical activity of acetic acid in DSF-induced culture media using K-ACET (Figure 7D). Compared to the positive control, the DSF group (2 μg/mL) induced a significant decrease in acetic acid content in the medium at 48 h (*p* < 0.01). This assay indirectly indicates that DSF diminishes the amount or activity of ALDH, thus inhibiting the ability of *C. neoformans* to metabolize aldehydes.

### 3.15 Homology modeling of CAT, AFR2, and ATM1 and molecular docking of DSF

**FIGURE 8:**
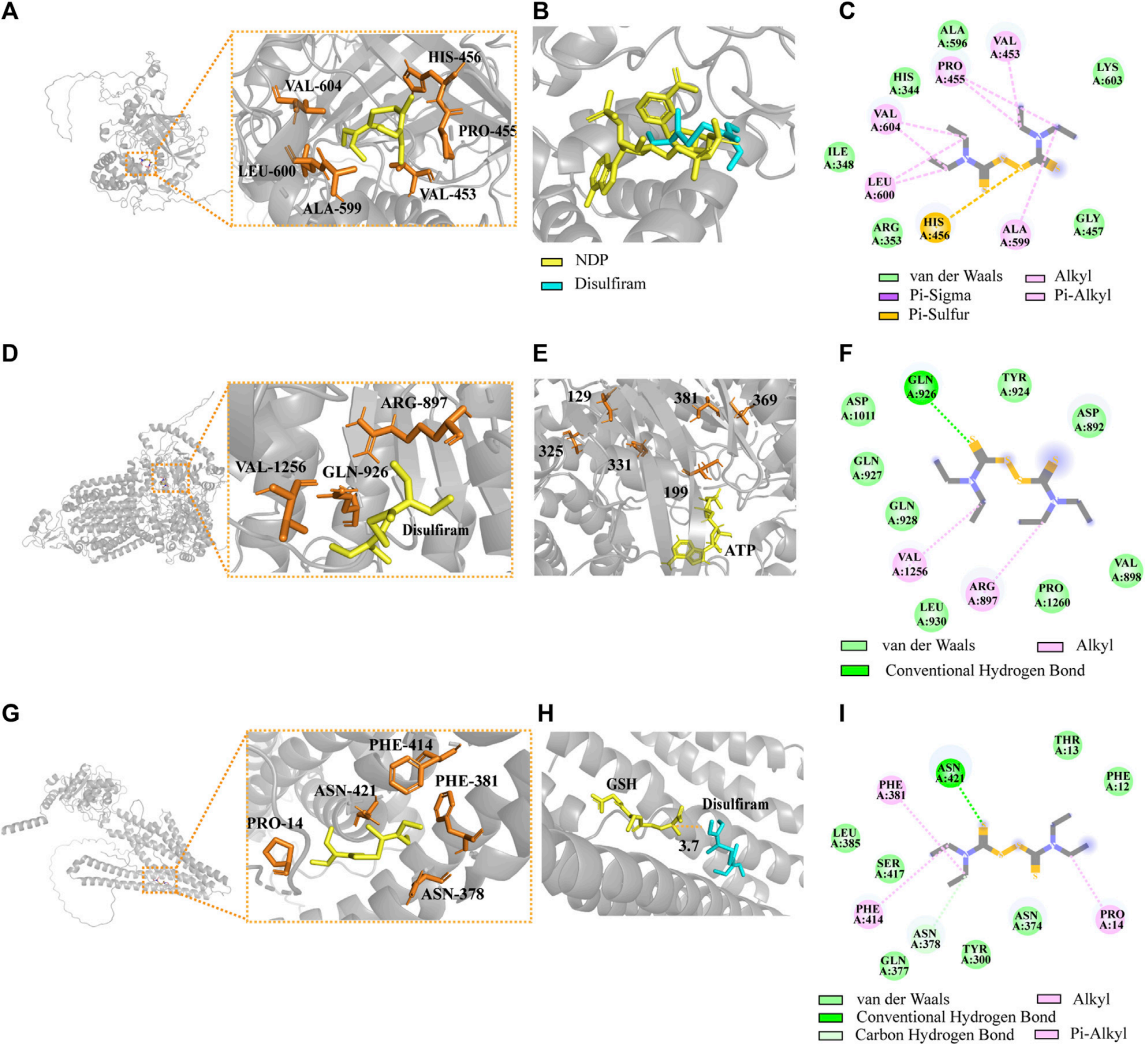
Complexes of the DSF ligand with predicted Alpha-Fold 3D structures of CAT, AFR2, and ATM1. **(A)** Structural models of CAT docking with DSF. **(B)** Introduction of the optimal DSF conformation for the homologous protein structure of CAT. **(C)** The 2D interactions between DSF and predicted binding sites of CAT. **(D)** Structural models of AFR2 docking with DSF. **(E)** Introduction of the optimal DSF conformation for the homologous protein structure of AFR2. **(F)** The 2D interactions between DSF and AFR2. **(G)** Structural models of ATM1 docking with DSF. **(H)** Introduction of the optimal DSF conformation for ATM1. **(I)** The 2D interactions between DSF and ATM1.

By combining CAT (UniProt ID: J9VNU0, ORF name: CNAG_05015), AFR2 (UniProt ID: J9VPA2, ORF name: CNAG_00869), and ATM1 (UniProt ID: J9VWU3, ORF name: CNAG_04358) of the Alpha-Fold conformation with DSF (Figures 8A, D, G), the optimal conformation binding affinities were −3.7, −3.8, and −3.8 kcal/mol, respectively. The best homologous protein to which CAT matched was the CAT of *Komagataella pastoris* (PDB ID 6RJN). The best docking conformation of DSF overlapped with the position of NDP (the active substrate of 6RJN), indicating that DSF successfully occupied the active NDP pocket (Figure 8B). The protein most homologous to AFR2 was derived from *S. cerevisiae S288C* (PDB ID 7P05). The best docking conformation of DSF, closing to Cys in the 1041th position of H99’s AFR2, was only 3.7 Å away from active substrate ADP of 7P05. Moreover, the ATP substrate was close to multiple active Cys residues, including the 129th, 199th, 325th, 331th, 369th, and 381th positions of 7P05 (Figure 8E), ranging from 6.9 to 11.5 Å. The most homologous protein (PDB ID: 4MYH) matching ATM1 of H99 was derived from *S288C*. The optimal docking conformation of DSF was only 3.7 Å away from the active substrate GSH position (Figure 8H).

## 4 Discussion and conclusion

Fungi pose a serious threat to humanity, with at least 300 million people suffering from severe fungal infections (Bongomin et al., 2017). In recent years, it has been found that patients coinfected with *C. neoformans* and SARS-CoV-2 are at a significant risk of death during the first 30 days. While new drugs are costly to develop and have long approval cycles, “Repurposed old drugs” bring new ideas and savings (Chong and Sullivan, 2007). To the best of our knowledge, this is the first systematic study assessing the effects of DSF on *Cryptococcus* spp.

In this study, 42 *Cryptococcus* strains of various serotypes were selected, all with excellent DSF MIC. This indicates that DSF has a broad-spectrum rather than individual inhibitory effect on *Cryptococcus*. Unfortunately, these strains did not include FLC-resistant strains. The combined sensitivity test revealed that DSF and AmB/5-FC/TRB/KET exerted synergistic inhibitory effects on *C. neoformans*. This suggests that DSF may possess pathways other than conventional antifungal drugs, or that there are unique targets that interfere with drug resistance. These combinations may reduce the toxic side effects of conventional antifungal agents.

The antimicrobial mechanism of DSF can be understood from three perspectives: synergism with copper, affinity for the cysteine mercapto group of target proteins, and immunomodulatory functions. Combined with the existing studies on DSF antifungals, this implies a focus on significant proteins containing cysteine near active sites associated with membrane transport function (especially copper) and redox. Transcriptome sequencing corroborated this hypothesis. Therefore, in this study, molecular docking calculations were performed between these important proteins and DSF based on computer simulations. Molecular docking calculations were used to assess the degree of interaction between the ligand and receptor (Morris and Lim-Wilby, 2008).

The inhibition of ALDH by DSF is the primary basis for its alcohol-withdrawal effect. DSF inhibits activated cysteine by forming a thiocarbamate-thiol structure, which irreversibly inhibits ALDH activity in the human liver. *In vitro*, the inhibition of hepatic mitochondrial ALDH is caused by the formation of intramolecular disulfide bonds between two of the three adjacent cysteines within the active site. *In vivo*, DSF can form a carbamoyl derivative with the Cys302 of ALDH (Shen et al., 2000). ALDH is an important metabolic enzyme in yeast. Throughout their growth, fungi are continuously subjected to various stresses; in particular, the metabolic products ethanol and acetaldehyde can accumulate to toxic levels (Ingram and Buttke, 1984; Jones, 1990). Yeasts use a specialized ALDH pathway to convert toxic aldehydes into less hazardous chemicals. By converting toxic byproducts into acetate, this pathway lowers the toxicity of byproducts and makes it easy for pathogens to adapt to their surroundings (Marchitti et al., 2008; Black et al., 2009).

ALDH in H99 showed significant downregulation (*p* < 0.05) in the KEGG Pathway significance analysis. To investigate how DSF affects cryptococcal ALDH activity at the molecular level, we aligned the target and homologous proteins. The distance between the homologous protein Cys302 and DSF was only 3.6 Å. At the same time, the Cys302 corresponded to the 356th Cys of H99 ALDH. The pocket where the DSF was located was also at the center of the adjacent Cys 96, 356, and 278 of H99 ALDH. In contrast, the spatial location of the optimally predicted DSF coincided with the human-derived ALDH active substrate, NAD^+^. This high similarity indicated that the disulfide bond of DSF reacted with cysteine in both ways, thereby affecting the functional activation of H99 ALDH.

Catalase (UniProt ID: J9VNU0, ORF name: CNAG_05015) is an important scavenger of reactive oxygen species in peroxisomes, protecting cells and lipids from the toxic effects of hydrogen peroxide (Gómez et al., 2019). CAT was significantly upregulated in the KEGG Pathway analysis. CAT interacts with DSF through six amino acid residues near the active pocket: VAL-604, HIS-456, PRO-455, VAL-453, ALA-599, and LEU-600 (Figure 8C). DSF overlaps with the active substrate NDP in the H99 CAT pocket, suggesting that DSF may structurally interfere with protein function. An earlier study also showed a significant reduction in catalase activity in the livers of rats fed high doses of DSF (1.0–4.5 g per kg/d) (Koivusalo, 1959). We hypothesized that during the early stages, DSF disturbs the redox homeostasis of H99 cells, leading to enhanced levels of intracellular oxidative stress and increased levels of free radicals, thus causing a compensatory negative feedback upregulation at the CAT transcriptome level. However, the exact regulatory mechanism remains to be explored.

DSF can reverse cerebellar degeneration related 1 (Cdr1)-mediated *Candida* resistance by interacting with the ATP/substrate-binding site of the transporter protein and forming a disulfide bridge (Prasad et al., 1995; Tsao et al., 2009). The functional region of AFR2 (845–1.087 bp), a multipotent drug-resistant efflux pump of H99, was highly similar to that of Cdr1 (859–1,103 bp) (Supplementary Figure S1). AFR2 (UniProt ID: J9VPA2; ORF name: CNAG_00869) was significantly downregulated in the transcriptome following DSF treatment. AFR2 interacts with DSF via ARG-897, GLN-926, and VAL1256 (Figure 8F). In the docking results, the six closest Cys near the ATP active site were only 6.9–11.5 Å from DSF (Sauna et al., 2004). DSF, within 8 Å from Cys, can interfere with ATP binding site activity by assisting cysteines to form intramolecular disulfide bonds. Whether this is related to the synergistic effect of DSF needs to be verified through further experiments.

The iron-sulfur cluster transporter ATM1 (UniProt ID: J9VWU3, ORF name: CNAG_04358) plays an important role in the production of cytoplasmic iron-sulfur proteins (Do et al., 2018). ATM1 depends on copper-dependent transcription factor 1 (CUF1), which functions in an environment of copper stress and increases susceptibility to copper toxicity (Garcia-Santamarina et al., 2017). Transcriptome analysis revealed that CUF1 was significantly downregulated. Studies have shown that DSF treatment decreases GSH levels, leading to increased oxidized glutathione (GSSG) levels, which interferes with the GSH/GSSG oxidoreduction state (Nagendra et al., 1994). ATM1 interacts with DSF via PHE-414, PHE381, ASN-2378, PRO-14, and ASN-421 in the active pocket (Figure 8I). DSF is only 3.7 Å away from the homologous protein substrate GSH. DSF consumes GSH and disrupts the oxidation-reduction environment, which may prevent GSH from forming an iron-sulfur conjugation complex with ATM1.

In conclusion, DSF possesses excellent *in vitro* and *in vivo* antifungal activity against *C. neoformans* and this effect is mediated through multiple mechanisms. DSF is an FDA-approved drug that can be repurposed as a potential treatment option for *C. neoformans*.

## Data Availability

The data presented in the study are deposited in the Gene Expression Omnibus repository, accession number GSE248987 (https://www.ncbi.nlm.nih.gov/geo/query/acc.cgi?acc=GSE248987).
