# Supplementary material for: Antifungal activity of the repurposed drug disulfiram against Cryptococcus neoformans
Source: Front Pharmacol. 2024 Jan 11;14:1268649. doi: 10.3389/fphar.2023.1268649 (PMC10808519; doi:10.3389/fphar.2023.1268649)
Supplement: Supplementary file 1 [file DataSheet1.doc]

Supplementary Material

# Supplementary Figures and Tables

## Supplementary Figures

**
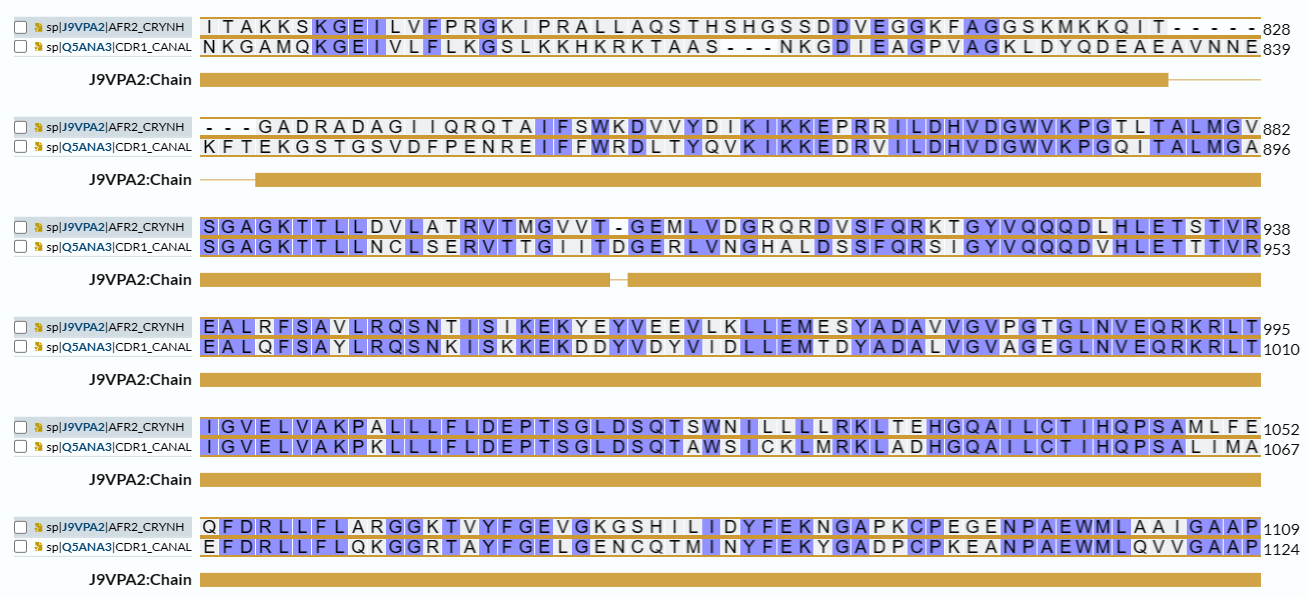
**

**Figure S1.** Comparison of partial functional region sequences of AFR2 (UniProt ID: J9VPA2) in *C. neoformans* H99 and Cdr1 (UniProt ID: Q5ANA3) in *Candida albicans*. By blasting the homologous genes of *Cdr1* in *C. neoformans* in the uniprot database (https://www.uniprot.org/), it was found that AFR2 (845-1087bp) of strain H99 has a high degree of similarity to the functional region (ABC transporter region) of CDR1 (859-1103bp). Among ABC transporters, Cdr1 has been shown to play a key role in azole resistance.


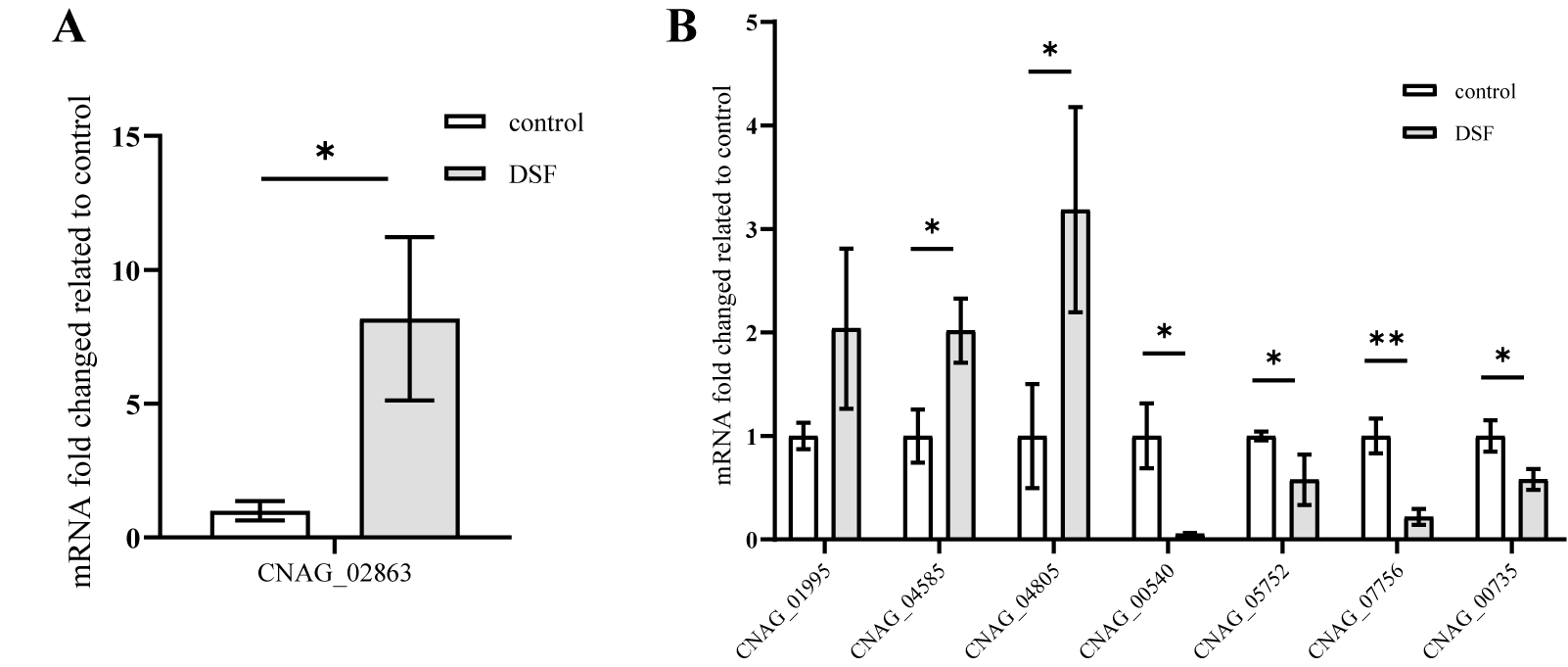


**Figure S2.** Eight genes that showed significant variability in transcriptome sequencing were identified by qpcr. (A) The transcriptome expression of *CNAG_04085* was significantly up-regulated in the DSF group compared to the control as measured by qpcr (*p* < 0.05). This is consistent with the transcriptome sequencing results. (B) The transcriptome expression of *CNAG_01995, CNAG_04585 and CNAG_02863* were significantly up-regulated in the DSF group, and the expression of *CNAG_05752, CNAG_07756, CNAG_00540, CNAG_00735* were significantly down-regulated. These results were consistent with transcriptome sequencing (**p* < 0.05, ** *p* < 0.01, vs. controls).

## Supplementary Tables

**Table A1** In vitro sensitivity assays of DSF and FLC against 42 strains of *Cryptococcus*^a^

| **Number** | **Strain** | **MIC(DSF)** | **MFC(DSF)** | **MIC(FLC)** |
| --- | --- | --- | --- | --- |
| 1 | D2A | 2.0 | 4.0 | 4.0 |
| 2 | ZYA2 | 2.0 | 4.0 | 8.0 |
| 3 | ZYA3 | 2.0 | 4.0 | 8.0 |
| 4 | ZYA4 | 4.0 | 4.0 | 8.0 |
| 5 | ZYA5 | 4.0 | 8.0 | 1.0 |
| 6 | ZYA6 | 2.0 | 4.0 | 4.0 |
| 7 | ZYA7 | 2.0 | 4.0 | 4.0 |
| 8 | ZYA8 | 2.0 | 4.0 | 2.0 |
| 9 | ZYA9 | 4.0 | 8.0 | 1.0 |
| 10 | ZYA10 | 4.0 | 8.0 | 0.5 |
| 11 | ZYA11 | 2.0 | 4.0 | 8.0 |
| 12 | ZYA12 | 4.0 | 4.0 | 1.0 |
| 13 | ZYA13 | 2.0 | 4.0 | 1.0 |
| 14 | B3501 | 2.0 | 8.0 | 2.0 |
| 15 | ZYA14 | 4.0 | 4.0 | 2.0 |
| 16 | ZYA15 | 8.0 | 32.0 | 2.0 |
| 17 | ZYA16 | 4.0 | 32.0 | 4.0 |
| 18 | ZYA17 | 8.0 | 16.0 | 4.0 |
| 19 | ZYA18 | 4.0 | 8.0 | 2.0 |
| 20 | ZYA19 | 4.0 | 8.0 | 1.0 |
| 21 | ZYA20 | 4.0 | 8.0 | 2.0 |
| 22 | ZYA21 | 2.0 | 16.0 | 4.0 |
| 23 | ZYA22 | 1.0 | 8.0 | 4.0 |
| 24 | ZYA23 | 4.0 | 16.0 | 4.0 |
| 25 | ZYA24 | 4.0 | 4.0 | 8.0 |
| 26 | ZYA25 | 4.0 | 4.0 | 8.0 |
| 27 | ZYA26 | 8.0 | 8.0 | 8.0 |
| 28 | ZYA27 | 4.0 | 4.0 | 1.0 |
| 29 | H99 | 4.0 | 8.0 | 2.0 |
| 30 | ATCC32608 | 4.0 | 4.0 | 4.0 |
| 31 | ZYB2 | 1.0 | 8.0 | 16.0 |
| 32 | ZYB3 | 8.0 | 32.0 | 4.0 |
| 33 | ZYB6 | 4.0 | 32.0 | 4.0 |
| 34 | ZYB8 | 8.0 | 16.0 | 1.0 |
| 35 | ZYB9 | 4.0 | 8.0 | 4.0 |
| 36 | ZYB10 | 4.0 | 16.0 | 2.0 |
| 37 | ZYB15 | 1.0 | 4.0 | 8.0 |
| 38 | ZYB24 | 2.0 | 4.0 | 8.0 |
| 39 | ZYB33 | 4.0 | 2.0 | 8.0 |
| 40 | ZYB34 | 2.0 | 32.0 | 8.0 |
| 41 | ZYB35 | 8.0 | 32.0 | 4.0 |
| 42 | ZYB65 | 8.0 | 16.0 | 2.0 |

^a^The number/strain, the DSF MIC determined by RPMI1640, FLC MICs, source of isolation or serotype of strains are indicated (MIC unit: μg/mL). MIC, minimum inhibitory concentration; MFC, minimal fungicidal concentration.

**Table A2 Checkerboard assay for DSF synergy with other antifungal drugs against B3501^a^**

| **Antifungal drug** | **Disulfiram**  **MIC alone** | **Antifungal drug**  **MIC alone** | **Disulfiram**  **MIC conbined** | **Antifungal drug MIC conbined** | **FICI** | **Indication** |
| --- | --- | --- | --- | --- | --- | --- |
| Amphotericin B | 2.0 | 0.5 | 0.25 | 0.125 | 0.375 | **Synergy** |
| Fluconazole | 2.0 | 2.0 | 0.25 | 1.0 | 0.625 | No interaciotn |
| 5-Fluorocytidine | 2.0 | 8.0 | 0.5 | 0.5 | 0.375 | **Synergy** |
| Terbinafine | 2.0 | 0.5 | 0.25 | 0.125 | 0.375 | **Synergy** |
| Ketoconazole | 2.0 | 0.125 | 0.25 | 0.03125 | 0.375 | **Synergy** |

^a^MIC, minimum inhibitory concentration (unit:μg/ml); FIC, fractional inhibitory concentration. The FICI model is calculated as FICI=FIC_A_+FIC_B_=[MIC_(A-combination)_/ MIC_(A-alone)_]+[MIC_(B-combination)_/MIC_(B-alone)_. FICI was interpreted as follows: ≤0.5, synergy; >4.0, antagonism; and 0.5<FICI≤4.0, no interaction.

**Table A3** Docking results of DSF with *C. neoformans* protein by AutoDock Tools 1.5.7.

| **Target protein** | **Uniprot ID** | **Optimal conformational binding energy(kcal/mol)** |
| --- | --- | --- |
| ALDH | J9VME7 | -4.4 |
| CAT | J9VNU0 | -3.7 |
| AFR2 | J9VPA2 | -3.8 |
| ATM1 | J9VWU3 | -3.8 |

**Table A4 Receptor homology modeling parameters^a^**

| **Target protein** | **Homologous protein PDB number** | **Sequence Identity** | **E-value** | **Region** | **RMSD(Å)** |
| --- | --- | --- | --- | --- | --- |
| ALDH | 4X0T | 51% | 2.279E-157 | 13-512 | 0.573  (2562 to 2562 atoms) |
| CAT | 6RJN | 52% | 4.717E-149 | 4-497 | 0.627  (2505 to 2505 atoms) |
| AFR2 | 7P05 | 48% | 0 | 36-1507 | 2.512  (8096 to 8096 atoms) |
| ATM1 | 4MYH | 59% | 2.719E-209 | 2-577 | 1.340  (3268 to 3268 atoms) |

^a^RMSD, root mean square deviation

**Table A5** Primer sequences^a^ of C. neoformans (H99) genes for quantitative RT-qPCR

| **Gene** | **Forward** | **Reverse** |
| --- | --- | --- |
| *CNAG_00735* | TCGTCCGCTTTCAACTCGTG | CCTCGACGCTGGCACTTTT |
| *CNAG_04085* | TTGGTCAATGTGAGTCGGGG | CTGCTCGCATTACTTTGCCG |
| | *CNAG_01995* | | --- | | TCCGCAGCAGTTAAAAGTCAA | AATGCCCGTGGCAGTGAC |
| *CNAG_05752* | CTAGAGCTGGTAGCGTCCTT | AGTCGTCCGTCATGGTCCTTTA |
| *CNAG_07756* | TTCCCATCCGCTTGGTTAGT | GGTTATAAATGGGTTGGAGACATC |
| *CNAG_00540* | TTGGGTTGCTGGTTCTCTGG | TATGAACAACCACCTCCAGCC |
| *CNAG_04585* | ACGATGGACGATGTGTTCGC | AGTGAGGGTATTATGCGCGTT |
| *CNAG_02863* | TTATCAGCGACCACTCCAGC | AGAGTTGTGCCCGAGTAATGG |
| *Actin* | ACACTGTCCCCATTTACGAAGG | CGGCAGAAGTGGTGAAGAGG |

^a^The primers referenced in this study were designed using the NCBI Primer BLAST system and purchased from SANGON (Shanghai, China).
